# A mouse model of acute exacerbations of lung inflammation in COPD with both steroid-sensitive and steroid-insensitive features

**DOI:** 10.1186/1476-9255-10-S1-P32

**Published:** 2013-08-14

**Authors:** V Russell, A Connolly, C Jagger, D Spicer, P Woodman, J Dlugozima, A Young

**Affiliations:** 1 Argenta Discovery, Spire Green, Harlow, Essex, UK

## Introduction

Exposure to tobacco smoke (TS) for 4 days induces a reproducible steroid-insensitive lung inflammation in the mouse. The effect of adding the viral mimetic poly IC (PIC) to TS-exposed mice was examined with the aim of defining the translational aspects of the model to human disease.

## Methods

Female mice (C57BL\6) were exposed daily to TS (Marlboro 100 cigarettes) for 4d. Control groups were exposed to air. Saline or PIC was administered intra-nasally. Mice were killed 4-120hrs after the last exposure, the lungs lavaged and cells (totals and differential) counted. Single oral doses of Dexamethasone (DEX 0.3mg/kg) or Roflumilast (ROF 5mg/kg) were administered 2hrs after the final challenge. To investigate the kinetics of the exaggerated inflammatory response, a single dose of DEX was given after the final challenge and effects followed for 120hrs.

## Results

TS-exposure caused a significant cellular infiltration (total cells ≈0.3 million) into the lung, which was inhibited by ROF but not DEX. In non-TS exposed mice, PIC induced an inflammatory response (total cells ≈0.3 million) which was not inhibited by either DEX or ROF. Administration of PIC in addition to TS-exposure induced an exaggerated inflammatory response (total cells ≈1.1 million) which was significantly greater than the additive effect of the two stimuli. This enhanced inflammatory response peaked 24hrs after the last exposure before slowly declining. Neutrophils were the predominant infiltrating cell type over the first 48hrs. Macrophage numbers were also elevated, with a sustained increase at 24-72hrs. Lymphocyte numbers also increased and peaked at 48-72hrs after exposure. The peak exacerbated inflammation at 24hrs after exposure was significantly inhibited by both ROF (53%, p<0.05) and DEX (56%, p<0.05), in contrast to the reproducible lack of efficacy of DEX in the TS or PIC groups. The time course revealed that a single dose of DEX given after the last exposure reduced the exaggerated response over the entire 120hr study period but was unable to fully resolve the inflammation.

## Conclusions

TS-exposure for 4 days induced a lung inflammation which was insensitive to steroids. Addition of PIC elicited a markedly enhanced inflammatory response that resolved over 120hrs and was sensitive to both steroids and Roflumilast, mimicking features of human COPD.

